# Proteasome inhibition reverses hedgehog inhibitor and taxane resistance in ovarian cancer

**DOI:** 10.18632/oncotarget.2295

**Published:** 2014-07-31

**Authors:** Adam D. Steg, Mata R. Burke, Hope M. Amm, Ashwini A. Katre, Zachary C. Dobbin, Dae Hoon Jeong, Charles N. Landen

**Affiliations:** ^1^ Department of Obstetrics and Gynecology, University of Alabama at Birmingham, Birmingham, AL; ^2^ McWhorter School of Pharmacy, Samford University, Birmingham, AL; ^3^ Institute of Oral Health Research, University of Alabama at Birmingham, Birmingham, AL; ^4^ Department of Obstetrics and Gynecology Busan Paik Hospital, Inje University, Gimhae, South Korea; ^5^ Department of Obstetrics and Gynecology, University of Virginia, Charlottesville, VA

**Keywords:** bortezomib, LDE225, paclitaxel, hedgehog, proteasome, ovarian cancer

## Abstract

The goal of this study was to determine whether combined targeted therapies, specifically those against the Notch, hedgehog and ubiquitin-proteasome pathways, could overcome ovarian cancer chemoresistance. Chemoresistant ovarian cancer cells were exposed to gamma-secretase inhibitors (GSI-I, Compound E) or the proteasome inhibitor bortezomib, alone and in combination with the hedgehog antagonist, LDE225. Bortezomib, alone and in combination with LDE225, was evaluated for effects on paclitaxel efficacy. Cell viability and cell cycle analysis were assessed by MTT assay and propidium iodide staining, respectively. Proteasome activity and gene expression were determined by luminescence assay and qPCR, respectively. Studies demonstrated that GSI-I, but not Compound E, inhibited proteasome activity, similar to bortezomib. Proteasome inhibition decreased hedgehog target genes (*PTCH1*, *GLI1* and *GLI2*) and increased LDE225 sensitivity *in vitro*. Bortezomib, alone and in combination with LDE225, increased paclitaxel sensitivity through apoptosis and G2/M arrest. Expression of the multi-drug resistance gene *ABCB1/MDR1* was decreased and acetylation of α-tubulin, a marker of microtubule stabilization, was increased following bortezomib treatment. HDAC6 inhibitor tubastatin-a demonstrated that microtubule effects are associated with hedgehog inhibition and sensitization to paclitaxel and LDE225. These results suggest that proteasome inhibition, through alteration of microtubule dynamics and hedgehog signaling, can reverse taxane-mediated chemoresistance.

## INTRODUCTION

Ovarian cancer is the leading cause of death from a gynecologic malignancy. Although ovarian cancer is among the most chemosensitive malignancies at the time of initial treatment (consisting of surgery and platinum/taxane-based chemotherapy), most patients will develop tumor recurrence and succumb to chemoresistant disease [1]. Evaluation of multiple chemotherapy agents in several combinations in the last 20 years has yielded modest improvements in progression-free survival, but no increase in durable cures. This clinical course suggests that multiple cellular pathways contribute to either inherent or acquired resistance to chemotherapy. Targeting these cellular pathways with combination therapies may provide better long-term outcomes if the chemoresistant population can be identified and targeted.

Cellular pathways normally involved in embryonic development, including Notch and hedgehog, have been found to be aberrantly expressed in a variety of malignancies [2-4], including ovarian cancer [5-11], and may be especially important in conferring resistance to chemotherapies [12-16]. Thus, inhibition of these pathways may offer valuable therapeutic strategies against ovarian cancer, either alone or as chemosensitizing agents. In particular, compounds that target gamma-secretase, which is crucial for Notch signaling activation [17], have been evaluated as potential anti-cancer agents. In addition, compounds have been developed that antagonize the Smoothened receptor, a mediator of hedgehog signaling [18]. One of these compounds, LDE225, has been used to target cancer cells in both pre-clinical and clinical models [18-21].

The ubiquitin-proteasome pathway is responsible for maintaining cellular homeostasis by regulating the degradation of proteins. Disruption of this pathway can result in cell cycle arrest and apoptosis as a result of incompatible regulatory protein accumulation within the cell [22]. Cancer cells generally have higher levels of proteasome activity and are more sensitive to the pro-apoptotic effects of proteasome inhibition than normal cells, making the proteasome a desirable therapeutic target [23]. Bortezomib is a dipeptidyl boronic acid-based reversible proteasome inhibitor that targets the chymotrypsin- and caspase-like active sites of the proteasome complex [24]. This compound was the first proteasome inhibitor to be approved for clinical use and is commonly used in the treatment of hematologic malignancies, including multiple myeloma and mantle cell lymphoma [25]. By inhibiting the proteasome, bortezomib acts through several mechanisms to suppress tumor survival pathways and to arrest tumor growth, metastasis and angiogenesis. These mechanisms of action have provided rationale for the combination of bortezomib with numerous chemotherapeutic and targeted agents [26-28], some of which have been evaluated in ovarian cancer clinical trials [29-31].

Novel therapeutic strategies targeting chemoresistant cells are essential to achieving durable cures in ovarian cancer. In our study, we sought to reverse resistance to chemotherapeutic and targeted agents using different pharmacological strategies. The results of this study demonstrate several novel mechanisms. First, the variable response seen with different gamma-secretase inhibitors is due to differential effects on the proteasome. Secondly, proteasome inhibition affects microtubule stabilization in a manner similar to taxanes and increases sensitivity to paclitaxel. Finally, proteasome inhibition alone reduces hedgehog pathway signaling and as a result is synergistic with hedgehog antagonist LDE225. The demonstrated crossover between these pathways sheds new light onto the contributing mechanisms of chemotherapy resistance in ovarian cancer and provides new opportunities for clinical development.

## RESULTS

### *In vitro* resistance to the Smoothened antagonist, LDE225, can be reversed by the gamma-secretase inhibitor GSI-I but not compound E

**Figure 1:**
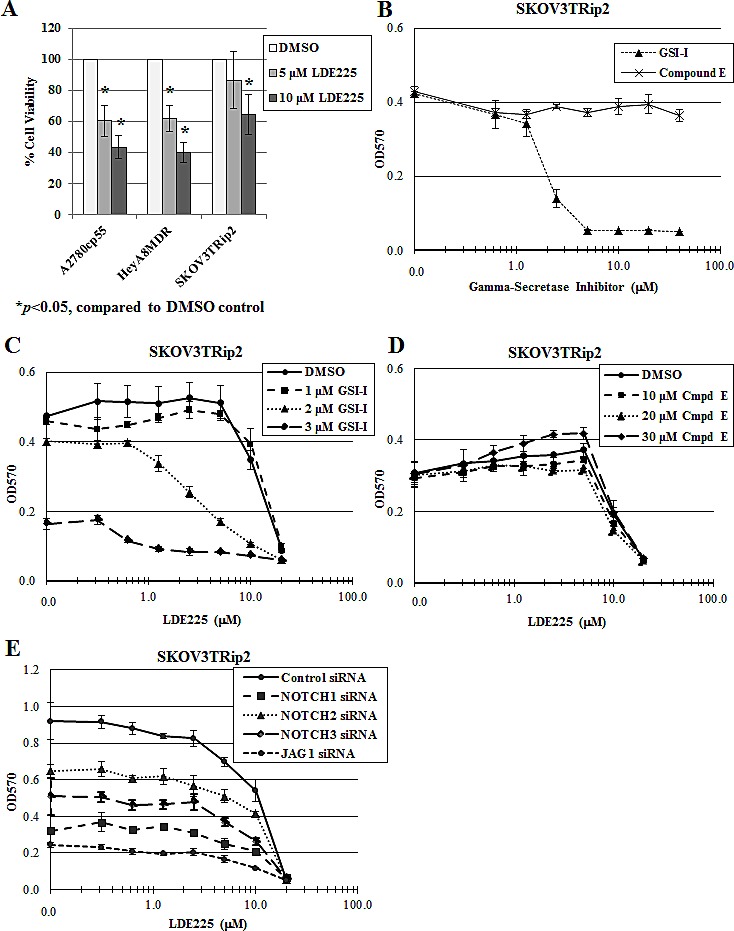
GSI-I, but not Compound E, reverses LDE225 resistance in SKOV3TRip2 cells A) Cell viability of chemoresistant ovarian cancer cell lines A2780cp55, HeyA8MDR and SKOV3TRip2 following exposure to the Smoothened antagonist, LDE225. B) SKOV3TRip2 cell viability in response to the gamma-secretase inhibitors, GSI-I and Compound E. C) SKOV3TRip2 cell viability following exposure to DMSO or GSI-I combined with increasing concentrations of LDE225. D) SKOV3TRip2 cell viability following exposure to DMSO or Compound E combined with increasing concentrations of LDE225. E) SKOV3TRip2 cell viability following knockdown of Notch signaling components (Notch1, Notch2, Notch3 and Jagged1) in combination with exposure to increasing concentrations of LDE225. In all experiments, cell viability was determined by MTT assay. Data are representative of at least 3 independent experiments.

We first sought to examine the mechanisms of dual inhibition of the Notch and Hedgehog pathways in three chemoresistant ovarian cancer cell lines: A2780cp55 (platinum- and taxane-resistant), HeyA8MDR (taxane-resistant) and SKOV3TRip2 (taxane-resistant). Dose-dependent growth inhibition with LDE225 alone is shown in Figure 1A. The decrease in A2780cp55 and HeyA8MDR cell viability following LDE225 treatment is similar (39.7% versus 38.2% decrease at 5 μM and 56.7% versus 60.1% decrease at 10 μM). However, SKOV3TRip2 cells responded to LDE225 to a lesser extent by comparison (13.5% and 35.4% decrease at 5 and 10 μM, respectively), suggesting that these cells have an innate mechanism of resistance to LDE225. Therefore, further combination strategies were pursued in this line in an attempt to uncover mechanisms of resistance to hedgehog inhibition.

Having previously demonstrated crosstalk between the Notch and Hedgehog pathways in SKOV3TRip2 cells [32], we wanted to determine if targeting the Notch pathway using gamma-secretase inhibitors could have an effect on response to LDE225 in these cells. To this end, we examined the effect of 2 different gamma-secretase inhibitors, GSI-I and GSI-XXI (Compound E) on the viability of SKOV3TRip2 cells. Interestingly, the viability of these cells was decreased following exposure to GSI-I, but not to Compound E (Figure 1B). Used in combination, GSI-I increased the sensitivity of SKOV3TRip2 cells to LDE225; up to a 17-fold decrease in the LDE225 IC50 compared to DMSO control was observed, suggesting a synergistic interaction (Figure 1C). Calculation of a combination index (CI=0.44 at 2μM, CI=0.11 at 3μM) confirms a synergistic effect. This effect was not observed with LDE225 in combination with Compound E (Figure 1D), suggesting that these gamma-secretase inhibitors may have differential mechanisms of action.

To determine if Notch inhibition is playing a role in LDE225 sensitization, knockdown of Notch signaling components (Notch1, Notch2, Notch3 and Jagged1) was carried out using siRNA. These siRNAs have previously been shown by our laboratory to decrease the mRNA levels of their respective target genes by up to 85% [32]. Alone, knockdown of these individual genes decreased SKOV3TRip2 cell viability (by 65.1%, 29.3%, 45.7% and 73.3%, respectively; p<0.05) compared to siRNA control, indicating that Notch signaling does contribute to the survival of these cells (Figure 1E). However, none of these siRNAs had a significant sensitizing effect on LDE225, as demonstrated by parallel dose response curves (Figure 1E) compared to the siRNA control. The fact that independent Notch family targeting and Compound E could not sensitize to hedgehog inhibition, as GSI-I could, suggest that the mechanism by which GSI-I sensitizes SKOV3TRip2 cells to LDE225 is independent of Notch inhibition.

### Proteasome inhibition reverses LDE225 resistance in SKOV3TRip2 cells

**Figure 2:**
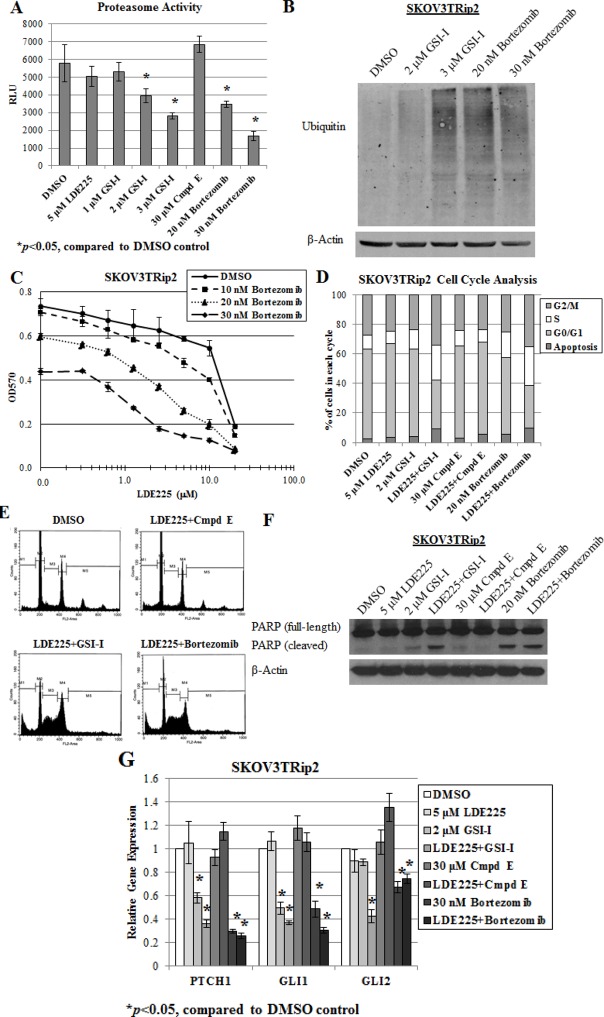
Proteasome inhibition reverses LDE225 resistance in SKOV3TRip2 cells A) Proteasome activity was measured in SKOV3TRip2 cells exposed to DMSO, LDE225, GSI-I, Compound E or bortezomib at the indicated concentrations for 24 hours. RLU = relative luminescence units. *P < 0.05, compared to DMSO vehicle control. B) Western blot analysis of ubiquitin was examined in SKOV3TRip2 cells exposed to DMSO, GSI-I or bortezomib overnight to detect the presence of polyubiquitinated proteins. β-actin was used as a loading control. C) SKOV3TRip2 cell viability following exposure to DMSO or bortezomib combined with increasing concentrations of LDE225, as determined by MTT assay. D) Cell cycle analysis was performed on SKOV3TRip2 cells treated with DMSO alone, LDE225 alone, GSI-I alone, Compound E alone, bortezomib alone, combined LDE225+GSI-I, combined LDE225+Compound E or combined LDE225+bortezomib for 72 hours using propidium iodide (PI) staining. E) Representative histograms of DMSO- and combination-treated cells are shown. F) Protein expression of PARP, an indcator of apoptosis, was examined in SKOV3TRip2 cells treated under the same conditions as those for PI staining using Western blot analysis. β-actin was used as a loading control. G) Gene expression of *PTCH1*, *GLI1* and *GLI2* was examined in SKOV3TRip2 cells treated with DMSO alone, LDE225 alone, GSI-I alone, Compound E alone, bortezomib alone, combined LDE225+GSI-I, combined LDE225+Compound E or combined LDE225+bortezomib for 24 hours. *P < 0.05, compared to DMSO vehicle control. Data are representative of at least 3 independent experiments.

Previous studies have demonstrated that GSI-I can act as a proteasome inhibitor [33-35]. To determine if GSI-I sensitizes SKOV3TRip2 cells to LDE225 through this mechanism rather than gamma-secretase inhibition, we first examined the effects of GSI-I, Compound E and bortezomib (a known proteasome inhibitor) on proteasome activity. In agreement with previous studies, both GSI-I and bortezomib produced a dose-dependent decrease in proteasome activity by up to 51.6% and 71.0%, respectively (p<0.05), whereas Compound E did not (Figure 2A). Moreover, treatment with GSI-I or bortezomib resulted in a significant increase in polyubiquitinated proteins, an indicator of proteasome inhibition [33] (Figure 2B). When combined with LDE225, bortezomib produced a similar synergistic effect on the viability of SKOV3TRip2 cells as that observed with GSI-I. Up to a 10-fold decrease in the LDE225 IC50 compared to DMSO control (CI=0.64 at 20nM, CI=0.38 at 30nM) was observed (Figure 2C).

To determine how proteasome inhibition combined with LDE225 might affect cell growth, we performed cell cycle analysis on SKOV3TRip2 cells that were treated with DMSO control, LDE225 (5 μM), GSI-I (2 μM), Compound E (30 μM), bortezomib (20 nM), or combined LDE225+GSI-I, combined LDE225+Compound E or combined LDE225+bortezomib for 72 hours. As shown in Figure 2D, LDE225+GSI-I and LDE225+bortezomib treatment combinations resulted in a greater accumulation of cells in the sub-G0/apoptotic (9.0% and 9.5%, respectively, versus 2.5% control), S (23.3% and 26.1%, respectively, versus 9.9% control) and G2/M (34.3% and 35.2%, respectively, versus 27.2% control) phases compared to DMSO control or either treatment alone (all p<0.05). Combined LDE225+Compound E did not have these effects. Representative flow cytometric graphs for combination therapy compared to control are shown in Figure 2E. In addition, poly (ADP-ribose) polymerase (PARP) cleavage, an indicator of apoptosis, was observed in SKOV3TRip2 cells treated with combined LDE225+GSI-I, bortezomib alone and combined LDE225+bortezomib (Figure 2F). These data suggest that the synergy between LDE225 combined with GSI-I or bortezomib results from cell cycle arrest at the S and G2/M phases, with the induction of apoptosis possibly playing a limited role.

To further examine the mechanism by which proteasome inhibition increases LDE225 sensitivity, we quantified gene expression of *PTCH1*, *GLI1* and *GLI2*, established markers of hedgehog pathway activity [36] in SKOV3TRip2 cells exposed to DMSO, LDE225 (5 μM), GSI-I (2 μM), Compound E (30 μM), bortezomib (30 nM), LDE225+GSI-I, LDE225+Compound E or LDE225+bortezomib after 24 hours using qPCR analysis. As shown in Figure 2G, LDE225-resistant SKOV3TRip2 cells demonstrated no significant decrease in *PTCH1*, *GLI1* or *GLI2* expression following exposure to single agent LDE225 (5 μM). This result agrees with LDE225′s lack of an effect on SKOV3TRip2 cell viability at this concentration (see Figure 1A). Surprisingly, GSI-I alone, but again not Compound E, led to a profound decrease in expression of *PTCH1* (by 41.8%, p<0.05) and *GLI1* (by 50.7%, p<0.05) compared to DMSO control. Moreover, combined GSI-I and LDE225 further decreased expression of *PTCH1*, *GLI1* and *GLI2* (by 64.2%, 63.2% and 57.6%, respectively; p<0.05) compared to DMSO control. Similar to GSI-I, bortezomib alone significantly decreased *PTCH1*, *GLI1* and *GLI2* expression in SKOV3TRip2 cells (by up to 70.2%, 51.6% and 32.9%, respectively; p<0.05) compared to DMSO control. Combined bortezomib and LDE225 further decreased expression of *PTCH1* and *GLI1* (by 74.2% and 69.4%, respectively; p<0.05) compared to LDE alone or DMSO control. These data establish a previously-unrecognized direct effect of proteasome inhibition on hedgehog signaling.

### Bortezomib decreases hedgehog transcriptional activity in ovarian cancer cell lines in a dose-dependent manner

**Figure 3:**
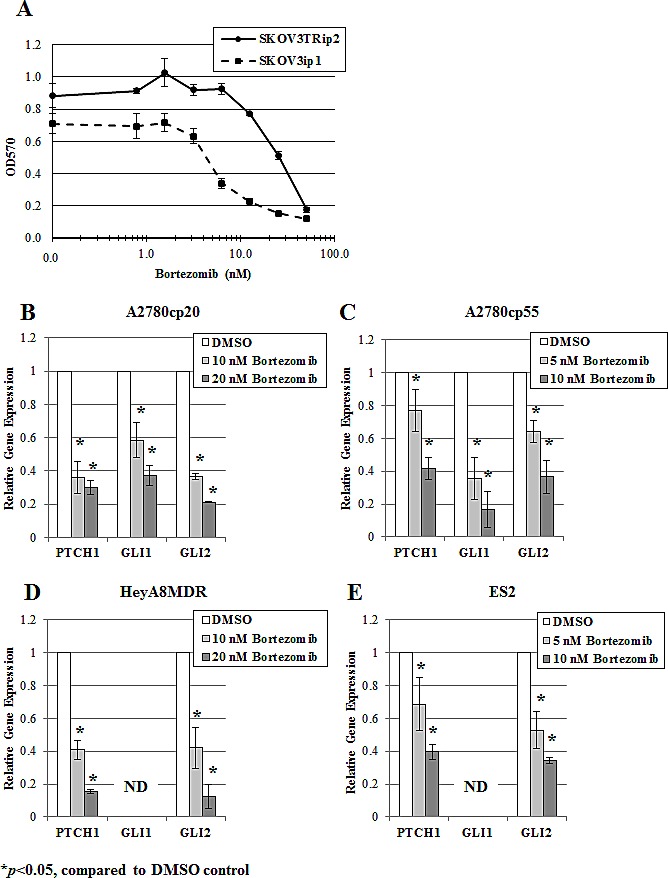
Bortezomib decreases hedgehog transcriptional activity in ovarian cancer cell lines in a dose-dependent manner A) SKOV3ip1 and SKOV3TRip2 cell viability following exposure to increasing concentrations of bortezomib, as determined by MTT assay. Gene expression of *PTCH1*, *GLI1* and *GLI2* was examined in B) A2780cp20, C) A2780cp55, D) HeyA8MDR and E) ES2 ovarian cancer cell lines treated with increasing concentrations of bortezomib for 24 hours, using quantitative PCR. ND = not detectable; *P < 0.05, compared to DMSO vehicle control. Data are representative of at least 3 independent experiments.

As shown in Table 1, OvCar3 and SKOV3TRip2 cells were the most sensitive and resistant, respectively, to GSI-I and bortezomib. Linear regression analysis of GSI-I and bortezomib response (IC50s) across all cell lines revealed a Pearson correlation coefficient (r) of 0.78, suggesting a similarity in drug response and mechanism of action (i.e. proteasome inhibition). SKOV3TRip2 cells demonstrated an increased resistance to both GSI-I and bortezomib compared to its parental, chemosensitive cell line, SKOV3ip1 (bortezomib viability results shown in Figure 3A). The effect of bortezomib on hedgehog transcriptional activity (as determined by *PTCH1*, *GLI1*, *GLI2* gene expression) in SKOV3TRip2 cells led us to evaluate this compound in other ovarian cancer cell lines. Interestingly, in all of the ovarian cancer cell lines that were examined by qPCR (A2780cp20, A2780cp55, HeyA8MDR, ES2), bortezomib significantly (p<0.05) decreased hedgehog transcriptional activity in a dose-dependent manner within 24 hours (Figure 3B-E). These results agree with the reductions in *PTCH1*, *GLI1* and *GLI2* expression observed in SKOV3TRip2 cells following bortezomib treatment (Figure 2E), further demonstrating crosstalk between the proteasome and hedgehog signaling pathways.

**Table 1:** GSI-I and bortezomib response in ovarian cancer cell lines

		
Cell Line	GSI-I IC_50_	Bortezomib IC_50_
A2780ip2	1.8 μM	7 nM
A2780cp20	1.7 μM	12.5 nM
A2780cp55	2.9 μM	8.5 nM
SKOV3ip1	1.2 μM	6 nM
SKOV3TRip2	3.1 μM	30 nM
HeyA8	2 μM	12 nM
HeyA8MDR	1.4 μM	9.5 nM
ES2	1.6 μM	3 nM
OvCar3	0.6 μM	1.5 nM

### Bortezomib increases paclitaxel sensitivity in chemoresistant ovarian cancer cells

**Figure 4:**
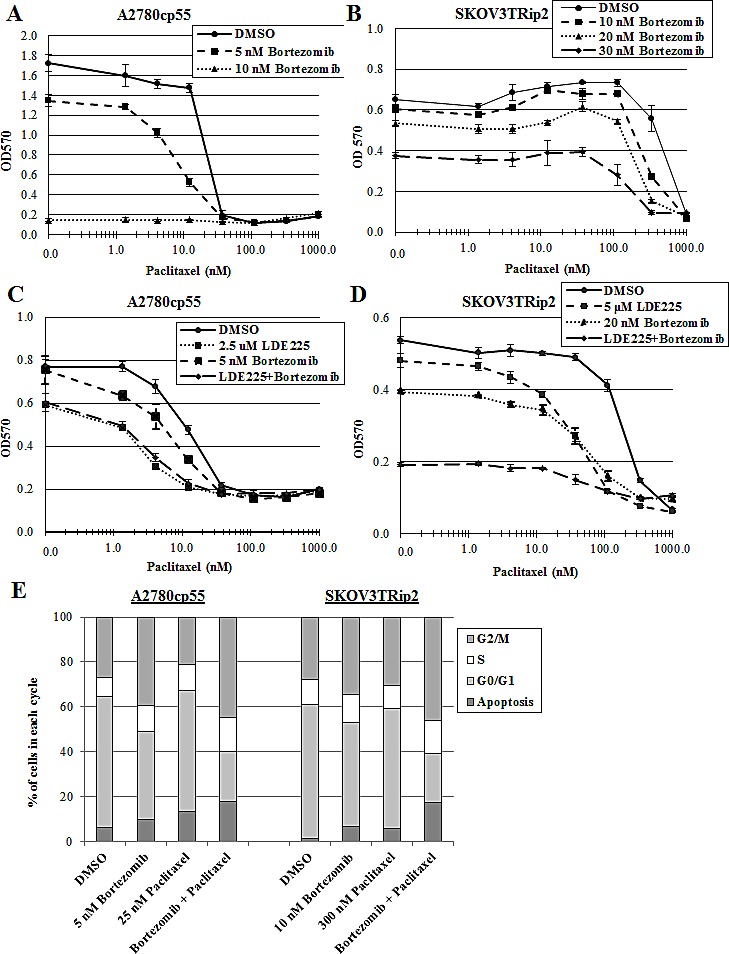

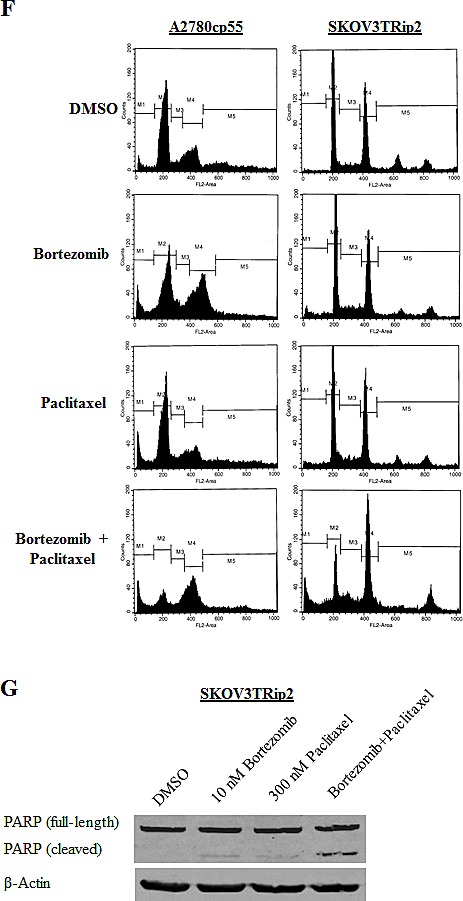
Bortezomib increases paclitaxel sensitivity in chemoresistant ovarian cancer cells A) A2780cp55 and B) SKOV3TRip2 cell viability following exposure to DMSO or bortezomib combined with increasing concentrations of paclitaxel. C) A2780cp55 and D) SKOV3TRip2 cell viability following treatment with DMSO alone, LDE225 alone, bortezomib alone or LDE225+bortezomib combined with increasing concentrations of paclitaxel. Cell viability was determined using MTT assay. E) Cell cycle analysis was performed on A2780cp55 and SKOV3TRip2 cells treated with DMSO alone, bortezomib alone, paclitaxel alone or combined bortezomib+paclitaxel for 72 hours using propidium iodide (PI) staining. F) Representative histograms of each treatment group in A2780cp55 and SKOV3TRip2 cells are shown. G) Protein expression of PARP, an indcator of apoptosis, was examined in SKOV3TRip2 cells exposed to DMSO alone, bortezomib alone, paclitaxel alone or combined bortezomib+paclitaxel for 72 hours using Western blot analysis. β-actin was used as a loading control. Data are representative of at least 3 independent experiments.

We have previously shown that antagonism of the hedgehog pathway, using LDE225 or siRNAs designed against hedgehog signaling components, can reverse taxane resistance in ovarian cancer cells [16]. The inhibitory effect of bortezomib on hedgehog signaling led us to consider whether this compound could be used to increase paclitaxel sensitivity. The chemoresistant ovarian cancer cell lines A2780cp55 and SKOV3TRip2 were exposed to DMSO or bortezomib combined with increasing concentrations of paclitaxel. Interestingly, we found that bortezomib combined with paclitaxel act in a synergistic manner. Up to a 2.3-fold decrease in paclitaxel IC50 compared to DMSO control was observed in A2780cp55 cells (CI=0.47) (Figure 4A) and up to a 2.6-fold decrease in paclitaxel IC50 compared to DMSO control was observed in SKOV3TRip2 cells (CI=0.41 at 30nM) (Figure 4B). To determine whether LDE225 combined with bortezomib could have a greater effect on paclitaxel sensitization, we exposed A2780cp55 and SKOV3TRip2 cells to DMSO, LDE225, bortezomib or LDE225+bortezomib combined with increasing concentrations of paclitaxel. In agreement with previous findings [16], LDE225 alone increased the sensitivity of A2780cp55 and SKOV3TRip2 cells to paclitaxel (Figure 4C, D); however, combined LDE225+bortezomib did not further enhance paclitaxel sensitization compared to single agents, indicating an additive effect. These results suggest that bortezomib can sensitize chemoresistant ovarian cancer cells to a similar degree as inhibition of hedgehog signaling.

To determine the mechanism through which bortezomib combined with paclitaxel might affect cell growth in a synergistic manner, we performed cell cycle analysis on A2780cp55 and SKOV3TRip2 cells after treatment with DMSO (vehicle control), bortezomib alone, paclitaxel alone or combined bortezomib+paclitaxel for 72 hours. As shown in Figure 4E/F, bortezomib+paclitaxel combination treatment resulted in a significantly greater (p<0.05) accumulation of cells in the sub-G0/apoptotic phase (17.9% versus 6.2%, 9.6%, 13.5% for A2780cp55; 17.1% versus 1.1%, 6.5%, 5.8% for SKOV3TRip2) and the G2/M phase (44.9% versus 27.0%, 39.4%, 21.3% for A2780cp55; 46.3% versus 27.8%, 34.6%, 30.3% for SKOV3TRip2) compared to DMSO control, bortezomib alone and paclitaxel alone, respectively. In addition, PARP cleavage was more readily observed in SKOV3TRip2 cells treated with combined bortezomib+paclitaxel compared to DMSO control and single agents (Figure 4G). Taken together, these data suggest that bortezomib, in combination with paclitaxel, induces apoptosis and cell cycle arrest at the G2/M phase.

### Modification of microtubule dynamics plays a role in the sensitization of paclitaxel and LDE225 by bortezomib

**Figure 5:**
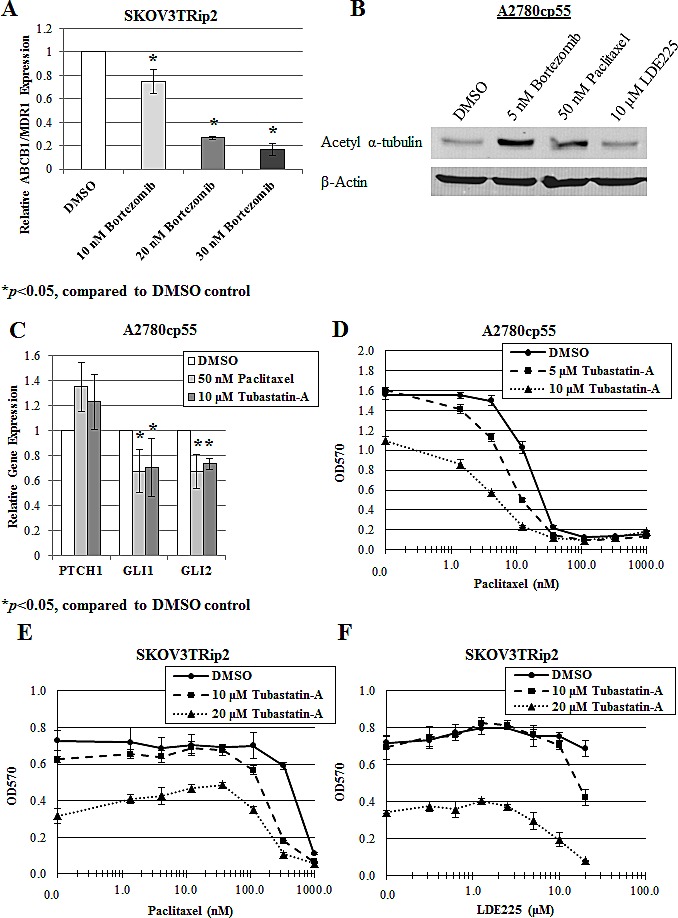
Modification of microtubule dynamics plays a role in the sensitization of paclitaxel and LDE225 by bortezomib A) Gene expression of *ABCB1/MDR1* was examined in SKOV3TRip2 cells treated with DMSO or bortezomib at the indicated doses for 72 hours, using quantitative PCR. *P < 0.05, compared to DMSO vehicle control. B) Protein expression of acetylated α-tubulin was examined in A2780cp55 cells treated with DMSO, bortezomib, paclitaxel or LDE225 for 24 hours using Western blot analysis. -actin was used as a loading control. C) Gene expression of *PTCH1*, *GLI1* and *GLI2* was examined in A2780cp55 cells treated with DMSO, paclitaxel or tubastatin-a for 24 hours, using quantitative PCR. *P < 0.05, compared to DMSO vehicle control. D) A2780cp55 and E) SKOV3TRip2 cell viability following exposure to DMSO or tubastatin-a combined with increasing concentrations of paclitaxel. F) SKOV3TRip2 cell viability following treatment with DMSO or tubastatin-a combined with increasing concentrations of LDE225. Cell viability was determined using MTT assay. Data are representative of at least 3 independent experiments.

To determine how inhibition of hedgehog signaling by bortezomib could promote increased paclitaxel sensitivity *in vitro*, we first examined expression of a primary mediator of taxane resistance *ABCB1/MDR1*. This gene encodes for P-glycoprotein, a drug efflux pump that has been shown by our laboratory to play a role in the synergy between LDE225 and paclitaxel [16]. Gene expression of *ABCB1/MDR1* was measured using qPCR after treatment with increasing concentrations of bortezomib for 72 hours. As shown in Figure 5A, bortezomib (10, 20, 30 nM) significantly (p<0.05) decreased *ABCB1/MDR1* expression in a dose-dependent manner (by 25%, 73% and 83%, respectively), indicating that bortezomib increases paclitaxel sensitivity, at least in part, through inhibition of drug efflux.

To more directly examine the effect of bortezomib on hedgehog signaling and chemotherapy response, we next evaluated the effect of bortezomib on microtubule stabilization. Recent studies have indicated that microtubule dynamics may play a role in bortezomib response [37, 38] as well as hedgehog signaling [39], thereby providing the rationale for examining this cellular process. A2780cp55 cells were treated with DMSO, bortezomib (5 nM), paclitaxel (50 nM) or LDE225 (10 M) for 24 hours, and examined for protein expression of acetylated α-tubulin, a marker of microtubule stabilization, using Western blot analysis. Treatment with bortezomib, and to a similar degree with paclitaxel, led to an increase in acetylated α-tubulin (Figure 5B). Alternatively, treatment with LDE225 did not have this effect, suggesting that hedgehog inhibition itself does not impact tubulin polymerization. An alternative relationship was hypothesized, whereby hedgehog signaling may actually be a downstream effect of microtubule stabilization. Indeed, A2780cp55 cells treated with microtubule-stabilizing agents, paclitaxel and the selective HDAC6 inhibitor, tubastatin-a [40], demonstrated modest but significant (p<0.05) decreases in *GLI1* (33% and 30%, respectively) and *GLI2* (33% and 26%, respectively) gene expression (Figure 5C), similar to decreases noted after treatment with bortezomib (Figure 2E, 3B-E). To determine whether microtubule effects play a role in drug sensitization, we exposed chemoresistant ovarian cancer cells to DMSO or tubastatin-a, combined with increasing concentrations of paclitaxel or LDE225. Similar to bortezomib, we found that tubastatin-a increased paclitaxel sensitivity in A2780cp55 cells (up to a 5-fold decrease in IC50 compared to DMSO control; Figure 5D) and in SKOV3TRip2 cells (up to a 3-fold decrease in IC50 compared to DMSO control; Figure 5E). Also similar to bortezomib, tubastatin-a increased LDE225 sensitivity in LDE225-resistant SKOV3TRip2 cells in a dose-dependent manner (Figure 5F). Taken together, these data suggest bortezomib can reverse taxane chemoresistance by interfering with microtubule dynamics and, subsequently, hedgehog pathway activity.

## DISCUSSION

In the current study, we found that inhibiting the proteasome, using two distinct pharmacologic agents, decreased hedgehog pathway activity and increased LDE225 sensitivity in an ovarian cancer model. Moreover, proteasome inhibition sensitized chemoresistant ovarian cancer cells to paclitaxel. These effects appear to be mediated, at least in part, by modification of microtubule dynamics within ovarian cancer cells. The participation of proteasome inhibition in reversing chemotherapeutic and targeted therapy resistance makes it an attractive clinical strategy, considering most ovarian cancer patients develop tumor recurrence and succumb to chemoresistant disease.

Recent studies have identified clinical resistance to selective hedgehog antagonists, such as GDC-0449/vismodegib and LDE225/erismodegib [18-21]; however, strategies for reversing this resistance have not been well defined. In a previous study, we reported that *GLI1* and *GLI2* mRNA levels, indicators of hedgehog signaling, were significantly higher in cancer cells isolated from persistent/chemoresistant ovarian tumors compared to those isolated from matched primary tumors [41]. In addition, we have shown that hedgehog signaling plays a role in ovarian cancer chemoresistance [16]. Based on these data, the utility of developing methods for increasing sensitivity to hedgehog antagonists becomes apparent. Initially, we focused on crosstalk between the hedgehog and Notch signaling pathways as a potential mechanism of LDE225 resistance, based upon the finding that the Notch ligand Jagged1 can regulate hedgehog signaling [32]. Inhibition of the gamma-secretase complex, which is essential for processing of the Notch signaling cascade, initially provided contrasting results, as one of the agents, GSI-I, sensitized ovarian cancer cells to LDE225, whereas another agent, GSI-XXI/Compound E, did not. Moreover, knockdown of individual Notch signaling components (Notch1, Notch2, Notch3 and Jagged1) did not increase LDE225 sensitivity, suggesting that Notch/hedgehog interactions are not involved in resistance to this compound. Since GSI-I, but not Compound E, was found to increase LDE225 sensitivity in ovarian cancer cells, we sought to identify an alternative mechanism of action for GSI-I that is independent of gamma-secretase inhibition. Recent studies have indicated that GSI-I can act as a proteasome inhibitor [33-35], largely due to the fact that this compound is derived from MG132, a known proteasome inhibitor [42]. Evaluation of GSI-I on proteasome activity revealed that this compound does significantly inhibit the proteasome in our ovarian cancer model.

Based on this evidence, we combined the more selective and clinically available proteasome inhibitor, bortezomib, with LDE225 to determine if the same sensitization would result, which we did observe *in vitro*. GSI-I and bortezomib also similarly increased cell cycle arrest (S and G2/M phases) and apoptosis in combination with LDE225, compared to DMSO control or single agents. It was demonstrated from the qPCR analysis of cells treated with GSI-I or bortezomib that there is crosstalk between the proteasome and hedgehog signaling pathways, as the mRNA levels of hedgehog target genes (*PTCH1*, *GLI1* and *GLI2*) decrease with exposure to either drug. It has been proposed that hedgehog inhibitor resistance may be due to three distinct mechanisms: 1) mutations within Smoothened that prevent molecular interaction with the inhibitor, 2) activation of compensatory pathways, 3) increased amplification of downstream mediators in the hedgehog pathway, such as the Gli transcription factors [18, 20]. If resistance to LDE225 is occurring upstream (perhaps at the level of Smoothened), it could be theorized that suppression of hedgehog signaling downstream of Smoothened (through proteasome inhibition) would help alleviate this resistance.

Based on our previous finding that hedgehog antagonism could be used to reverse taxane resistance [16], we asked whether proteasome inhibition, through its effect on hedgehog, could be used to achieve the same goal. Indeed, we found that bortezomib sensitized chemoresistant ovarian cancer cells to paclitaxel. This combination effect was accompanied by decreased expression of *ABCB1/MDR1*, a drug efflux pump that is strongly associated with taxane resistance and hedgehog pathway upregulation [14, 16]. Having found that bortezomib was able to increase sensitivity to LDE225 and paclitaxel *in vitro*, we sought to identify a common mechanism that could account for these biologic effects. Recent studies have implicated microtubule stabilization as one of bortezomib's mechanisms of action [37, 38]. In addition, microtubule dynamics have been shown to play a role in regulating hedgehog signaling [39]. These studies led us to investigate the impact of bortezomib on microtubule function. In agreement with microtubule-stabilizing agents, bortezomib alone, and in combination with paclitaxel, was found to induce G2/M phase arrest. Moreover, bortezomib increased acetylation of α-tubulin, a marker of microtubule stabilization and HDAC6 inhibition [43], compared to DMSO control. As single agents, the microtubule stabilizing agents, paclitaxel and the selective HDAC6 inhibitor, tubastatin-a, decreased hedgehog gene expression, suggesting that the changes in microtubule dynamics following bortezomib treatment are associated with decreases in hedgehog signaling. LDE225 did not affect acetylated α-tubulin, indicating that hedgehog inhibition itself is not responsible for microtubule stabilization, but rather occurs downstream of this event. Sensitization of chemoresistant ovarian cancer cells to paclitaxel and LDE225 by tubastatin-a further suggests that microtubule effects are a mechanism whereby bortezomib can induce chemosensitivity.

Because bortezomib and paclitaxel seem to share a common mechanism of action (i.e. microtubule stabilization), it would seem counterintuitive for these agents to act in a synergistic manner when combined. The same could be said of the combination effects of tubastatin-a and paclitaxel. As suggested by Poruchynsky et al. [37], it could be postulated that proteasome inhibition increases the expression of proteins that help stabilize microtubules, thereby providing a favorable environment for paclitaxel effects. In addition, HDAC6 may play a role in regulating these microtubule-associated proteins or vice versa, thereby affecting microtubule dynamics in a manner independent of the mechanism by which paclitaxel causes microtubule stabilization, which is through direct binding to tubulin [44]. In terms of LDE225 sensitization, it could be additionally inferred that HDAC6 inhibition results in decreased hedgehog signaling downstream of Smoothened, thereby overcoming potential resistance mechanisms that exist at the level of this receptor or compensatory pathways.

Collectively, this study validates proteasome inhibition as a strategy for reversing resistance to chemotherapy and hedgehog-targeting agents in an ovarian cancer model. With the ability to identify cancer patients whose tumors have active hedgehog signaling, proteasome inhibition could ultimately provide a useful therapeutic strategy for reversing chemoresistance and increasing overall survival in ovarian cancer patients.

## METHODS

### Reagents and cell culture

LDE225 was kindly provided by Novartis Pharma AG (Basel, Switzerland) and dissolved in DMSO to create a 10 mM stock solution. Gamma-secretase inhibitor I (GSI-I; Calbiochem, Billerica, MA) and gamma-secretase inhibitor XXI (Compound E; Calbiochem) were each dissolved in DMSO to create 4 mM stock solutions. Bortezomib (Selleckchem, Houston, TX) was dissolved in DMSO to prepare a 10 mM stock solution. Tubastatin-A (Sigma-Aldrich, St. Louis, MO), a kind gift of Dr. Douglas R. Hurst, was dissolved in DMSO to prepare a 5 mg/ml (13.5 mM) stock solution. The ovarian cancer cell lines A2780ip2, A2780cp20, A2780cp55, ES2, HeyA8, HeyA8MDR, OvCar3, SKOV3ip1 and SKOV3TRip2 were maintained in RPMI-1640 medium supplemented with 10% fetal bovine serum (Hyclone, Logan, UT). A2780cp20 and A2780cp55 (platinum- and taxane-resistant), HeyA8MDR (taxane-resistant) and SKOV3TRip2 (taxane-resistant, a kind gift of Dr. Michael Seiden [45]) were generated by sequential exposure to increasing concentrations of chemotherapy [46]. HeyA8MDR and SKOV3TRip2 were maintained with the addition of 150 ng/ml of paclitaxel. All cell lines were routinely screened for *Mycoplasma* species (GenProbe detection kit; Fisher, Itasca, IL) with experiments performed at 70-80% confluent cultures. Purity of cell lines was confirmed with STR genomic analysis, and only cells less than 20 passages from stocks were used in experiments.

### Cell viability assays

In each well of a 96-well plate, 2,000 cells were exposed to increasing concentrations of single agents, in triplicate. For combination studies, cells were treated with fixed doses of GSI-I, Compound E, bortezomib or tubastatin-a combined with increasing concentrations of LDE225, in triplicate. In addition, cells were exposed to fixed doses of bortezomib, tubastatin-a, LDE225 or LDE225+bortezomib combined with increasing concentrations of paclitaxel, in triplicate. For combination studies, all drugs were added at the same time and cells were allowed to grow until the control groups reached 80-90% confluency (usually 72 hours). After this time, 50 μL of 0.15% MTT dye (Sigma-Aldrich) was added to each well and the plate was incubated at 37°C for 2 hours. Conversion of MTT to formazan, a measure of cell viability, was determined using an Epoch Microplate Spectrophotometer (BioTek, Winooski, VT). IC50 of the drug of interest was determined by finding the dose at which 50% inhibition of cell viability was achieved, calculated by the equation [(OD570_MAX_-OD570_MIN_)/2) + OD570_MIN_].

### siRNA transfection

To examine the effect of Notch1, Notch2, Notch3 or Jagged1 knockdown on LDE225 response *in vitro*, SKOV3TRip2 cells were exposed to control siRNA (target sequence: 5′-UUCUCCGAACGUGUCACGU-3′, Sigma-Aldrich), NOTCH1-targeting siRNA (5′-GUGUGAAUCCAACCCUUGU-3′, Sigma-Aldrich), NOTCH2-targeting siRNA (5′-CUGUCAUACCCUCUUGUGU-3′, Sigma-Aldrich), NOTCH3-targeting siRNA (5′-GGUAGUAAUGCUGGAGAUU-3′, Sigma-Aldrich) or JAG1-targeting siRNA (5′-CCUGUAACAUAGCCCGAAA-3′, Sigma-Aldrich) at a 1:3 siRNA (μg) to Lipofectamine 2000 (Invitrogen, Carlsbad, CA) (μL) ratio. These siRNAs have previously been validated as having decreased the mRNA and protein levels of their respective target [32]. Cells were first transfected with siRNA (5 μg) overnight in 6-well plates (250, 000 cells/well), then trypsinized and re-plated at 2,000 cells per well on a 96-well plate, followed by addition of increasing concentrations of LDE225 after attachment. Cell viability was then assessed by MTT assay.

### Proteasome activity assay

SKOV3Trip2 cells were plated in white-walled 96-well plates (2,000 cells/well), allowed to attach overnight, and exposed to DMSO, LDE225, GSI-I, Compound E or bortezomib, in quadruplicate, at the indicated doses for 24 hours. Proteasome activity was measured in these treated cells using the Proteasome-Glo Chymotrypsin-Like Cell-Based Assay kit (Promega, Madison, WI). One hundred μl of Proteasome-Glo reagent was added to the 100 μl of media present in each well, mixed for 2 minutes at 700 rpm using a plate shaker and incubated at room temperature for 2 hours. Luminescence was measured in each well using a Synergy HT Microplate Reader (Biotek).

### Cell cycle analysis

Cells were treated with the indicated agents for 72 hours, trypsinized, and fixed in 100% ethanol overnight at 4°C. Cells were then centrifuged, washed in PBS, and resuspended in PBS containing 0.1% Triton X-100 (v/v), 200 μg/mL DNase-free RNase A and 20 μg/mL propidium iodide (PI). Cells were incubated in the PI staining solution for at least 30 minutes at 4°C prior to analysis. PI fluorescence was assessed by flow cytometry and the percentage of cells in sub-G0, G0/G1, S and G2/M phases was calculated by the cell cycle analysis module for Flow Cytometry Analysis Software (FlowJo v.7.6.1, Ashland, OR).

### RNA extraction and reverse transcription

Total RNA was isolated from ovarian cancer cell lines using Trizol reagent (Invitrogen) per manufacturer's instructions. RNA was then DNase treated and purified using the RNeasy Mini Kit (QIAGEN, Hilden, Germany). RNA was eluted in 50 μL of RNase-free water and stored at -80°C. The concentration of all RNA samples was quantified by spectrophotometric absorbance at 260/280 nm using a Take3 Micro-Volume Plate in an Epoch Microplate Spectrophotometer (BioTek). Prior to cDNA synthesis, all RNA samples were diluted to 20 ng/μL using RNase-free water. cDNA was prepared using the High Capacity cDNA Reverse Transcription Kit (Applied Biosystems, Foster City, CA). The resulting cDNA samples were analyzed using quantitative PCR.

### Quantitative PCR

Primer and probe sets for *GLI1* (Hs00171790_m1), *GLI2* (Hs00257977_m1), *ABCB1*/*MDR1* (Hs00184500_m1), *PTCH1* (Hs00181117_m1) and *RPLP0* (Hs99999902_m1; housekeeping gene) were obtained from Applied Biosystems and used according to manufacturer's instructions. PCR amplification was performed on an ABI Prism 7900HT sequence detection system and gene expression was calculated using the comparative C_T_ method as previously described [47]. Briefly, this technique uses the formula 2^−ΔΔC^_T_ to calculate the expression of target genes normalized to a calibrator. The cycling threshold (C_T_) indicates the cycle number at which the amount of amplified target reaches a fixed threshold. C_T_ values range from 0 to 40 (the latter representing the default upper limit PCR cycle number that defines failure to detect a signal).

### Western blot analysis

Cell lysates were collected in modified radioimmunoprecipitation assay (RIPA) lysis buffer with protease inhibitor cocktail (Roche, Manheim, Germany). Lysates were subjected to immunoblot analysis by standard techniques [46] using ubiquitin antibody (P4D1, Cell Signaling Technology, Danvers, MA), poly (ADP-ribose) polymerase (PARP) antibody (7D3-6, BD Pharmingen, San Jose, CA), or acetylated α-tubulin antibody (D20G3, Cell Signaling Technology) at 1:1000 dilution overnight at 4°C or anti-β-actin antibody (AC-15, Sigma-Aldrich) at 1:10,000 dilution for 1 hour at RT, which was used to monitor equal sample loading. After washing, blots were incubated with goat anti-rabbit (for acetylated α-tubulin) or goat anti-mouse (for ubiquitin, PARP, β-actin) secondary antibodies (Bio-Rad, Hercules, CA) conjugated with horseradish peroxidase. Visualization was performed by the enhanced chemiluminescence method (Pierce Thermo Scientific, Rockford, IL).

### Statistical analysis

Comparisons of cell viability, gene expression, relative luminescence units (RLU), and PI fluorescence were analyzed using a two-tailed Student's t-test, if assumptions of data normality were met. Those represented by alternate distribution were examined using a nonparametric Mann-Whitney U test. In the case of multiple group comparisons, one-way analysis of variance (ANOVA) followed by Tukey's post-test was performed. Differences between groups were considered statistically significant at p<0.05. Error bars represent standard deviation unless otherwise stated. The combination index (CI) method [48] was used to identify synergistic interactions between different compounds in their effect on cancer cell viability. A CI value equal to 1 indicates an additive effect, a CI value less than 1 indicates synergy (greater than additive) and a CI value greater than 1 indicates antagonism.
